# Class-Level School Performance and Life Satisfaction: Differential Sensitivity for Low- and High-Performing School-Aged Children

**DOI:** 10.3390/ijerph15122750

**Published:** 2018-12-05

**Authors:** Katharina Rathmann, Max Herke, Ludwig Bilz, Arja Rimpelä, Klaus Hurrelmann, Matthias Richter

**Affiliations:** 1 Department of Nursing and Health Science, University of Applied Sciences, 36037 Fulda, Germany; 2 Department for Sociology of Rehabilitation, Faculty of Rehabilitation Sciences, Technical University Dortmund, 44227 Dortmund, Germany; 3 Institute of Medical Sociology, Medical Faculty, Martin Luther University Halle-Wittenberg, 06112 Halle (Saale), Germany; max.herke@medizin.uni-halle.de (M.H.); m.richter@medizin.uni-halle.de (M.R.); 4 Department of Health Sciences, Faculty for Health, Social Work, and Music, Brandenburg University of Technology Cottbus-Senftenberg, 01968 Senftenberg, Germany; ludwig.bilz@b-tu.de; 5 Faculty of Social Sciences, Health Sciences and PERLA (Tampere Center for Childhood, Youth and Family Research), University of Tampere, 33014 Tampere, Finland; Arja.Rimpela@uta.fi; 6 Tampere University Hospital, Department of Adolescent Psychiatry, 33380 Pitkäniemi, Finland; 7 Hertie School of Governance, 10117 Berlin, Germany; hurrelmann@hertie-school.org

**Keywords:** school performance, social comparison, life satisfaction, “big-fish-little-pond”-effect, multilevel analysis, National Educational Panel Study, Germany

## Abstract

This study investigates whether class-level school performance affects students’ life satisfaction and if there are differential effects for high- and low-performing students. Data were derived from the National Educational Panel Study, including n = 5196 students (49.6% girls), nested in 478 classes and 250 secondary schools. School performance in class was measured by aggregating individual grade point average in Mathematics and German. The study could not reveal the “big-fish-little-pond”-effect regarding students’ life satisfaction but found differential effects for high- vs. low performing students. There was no significant association for low-performing students attending classes with higher class-level performance However, low-performing students revealed the lowest life satisfaction. High-performing students placed in classes with higher average performance reported lower life satisfaction compared to high-performing students in classes with lower average performance. This study provides evidence for the impact of the learning environment in class on school-aged children’s life satisfaction, by highlighting the differential sensitivity of high-performing students when placed in classes with higher or lower average performance.

## 1. Introduction

Adolescence is a vulnerable stage in life when young people become increasingly self-conscious and more aware of and concerned with others’ opinions, while peers become increasingly important. Already in school, children compare themselves with others and understand that others are making comparisons and judgments about them; they also begin to place higher value on these judgments [1]. In this regard, comparisons with reference groups are typical processes to shape adolescents’ self-perception and -evaluation. Particularly classrooms are peculiar contexts, where young people are confronted with reference group comparisons between classmates in terms of social or scholastic issues [2,3,4,5]. For instance, comparisons of abilities and school grades with peers and classmates in adolescence are associated with young people’s general and academic self-concept [6] as well as overall well-being [7]. This implies that peers in the class also function as reference groups for students to form their academic self-evaluation and self-concept [8,9,10]. In general, self-concept refers to individuals’ self-evaluation of their own abilities, substantially developed through comparisons with others [11,12], while academic self-concept refers to their self-evaluation regarding a specific academic domain or ability [13,14].

### 1.1. The Big-Fish-Little-Pond (BFLP)- or Contrast-Effect

Among the most important influences on students’ academic self-concept are reference group effects or social comparisons, in which students compare their self-perceived performance or school grades with the perceived performance of others within a particular achievement domain [15,16,17]. Further, it is also likely that social comparisons cause students to question their academic competences, to notice that their self-esteem is being threatened and probably make them evaluate their school performance as less competent in relation to peers [6]. Self-esteem is related to self-concept, but unique in its own right, it represents the overall emotional evaluation of one’s self-worth or a value judgment of oneself [18].

Most prominent in relation to social comparisons between students in educational settings, studies have highlighted that equally able students have lower academic self-concepts when attending schools or classes where the average ability level of classmates is higher, and higher academic self-concepts when attending schools or classes where the school- or class-average ability is lower [7,8,9,10,19,20]. This association is well-known as the so-called “contrast-” or “big-fish-little-pond”-effect (BFLPE). According to the BFLPE-hypothesis, it is argued that it is better for school-aged children’s general or academic self-concept to be a “big fish” in a “little pond” (i.e., in reference groups or classes with on average lower performance levels) than to be a “little fish” in a “big pond” (i.e., in reference groups or classes with on average higher performance levels). In other words, a student will have a lower (academic) self-concept (“little fish”) in a learning group with higher school performance (“big pond”) and will have a higher (general or academic) self-concept (“big fish”) in a class with lower school performance (“little pond”) because this student compares and contrasts his or her own school performance with that of classmates. Empirical support for this contrast- or BFLP-effect comes from manifold national and cross-national studies, for different educational settings [17,19,21,22] and mainly in relation to young people’s general or academic self-concept as well as self-esteem [9]. Findings on the BFLPE are remarkably robust, generalizing over a wide variety of different individual student and contextual level characteristics, settings, countries, long-term follow-ups, and research designs [17,20,23]. In sum, the BFLPE is analyzed in prior studies, by using students’ individual performance and class-level performance as the average or mean-value of class performance [see for example: 8]. However, studies using other outcomes of students’ well-being, such as general life satisfaction when examining the BFLPE, are not available, so far.

### 1.2. Correlates of Life Satisfaction

Although manifold studies have focused on self-concept or self-esteem, these are concepts which only represent one facet of young people’s psychosocial development and well-being [7,19,24]. In general, the concept of life satisfaction refers to evaluative aspects of well-being in the sense that an individual judges his or her position in life [25]. It is one of three main components of well-being (life satisfaction, positive affect and negative affect) [26], and reflects an overall evaluation of well-being [27]. Thus, life satisfaction forms a component of well-being and is often used synonymously for well-being [28]. Regarding young people’s psychosocial development, life satisfaction is an important and stable indicator of well-being as it reflects the match between students’ developmental needs and the social environment [24,29], such as schools [3]. Life satisfaction as an evaluation of an individual’s quality of life is closely linked to subjective health [30], social competence and good coping skills [31]. Prior studies revealed that life satisfaction is not only an important predictor of life outcomes in adulthood, but it is also important in predicting the development of young people [32,33,34,35].

So far, many studies have shown that life satisfaction is associated with a number of other positive outcomes, for example mental health [36], positive attitudes towards life, or self-esteem [24]. With regard to school life, self-reported experiences at school are closely related to young people’s life satisfaction [37,38,39]. For instance, higher performance—measured by subjective indicators of self-evaluated or perceived academic performance—is strongly correlated with higher life satisfaction, whereas the association between objective measures of academic achievement (i.e., achievement test scores) and life satisfaction is less clear [40,41]. In sum, previous evidence mainly focused on individual-level associations between perceived class climate [42] or indicators of students’ performance and life satisfaction.

### 1.3. The Importance of the Class Environment

As research findings above have illustrated, prior studies have attempted to explain students’ life satisfaction mostly by individual perceptions of class climate features, which have been reported by students. However, from a theoretical point of view, school is considered as a multilevel phenomenon [43]—meaning that students are embedded in classes and classes are nested in schools which also have their specific peculiarities—it is quite plausible that not only the individual performance is important for young people’s life satisfaction, but also the social context, in which young people learn [3]. In this context, the school and class context is important as well, contributing to young people’s life satisfaction as class and school mates share a certain learning environment during school days [38]. 

Prior research work on the BFLPE examined class- and school-level achievement in relation to general or academic self-concept in order to test different frames of reference, either in class or school [44]. This study revealed that the school-level average performance has no effect on students’ self-concept after having controlled for class-level average performance, indicating that the more proximal frame of reference in class (i.e., local dominance) is more closely related to students’ self-concept compared to the more distal frame of reference of the school. According to these findings and the frame of reference theory, classrooms constitute the most important psychosocial environment of educational settings for young people in terms of the learning climate, student cooperation, competition, student participation and school engagement, but also in terms of shared beliefs, emotions, habits and peer pressure, also having an impact on student life satisfaction in both positive and negative ways [45]. Particularly, adolescence is a phase when young people are required to fulfill a variety of developmental tasks, particularly in schools and with peer groups. 

According to the theory of the stage-environment-fit [2], mismatches between the learning environment in class and the developmental needs and capacities of students may have a negative impact on their overall satisfaction with life or psychosomatic health of students [45]. Given the amount of time adolescents spend in class, there is a particular need for further research investigating young people’s life satisfaction in relation to the average performance of the class. 

Regarding the contrast-effect of the BFLPE-hypothesis, the notion of relative deprivation could be closely linked to young people’s development [1] and life satisfaction in particular, as young people make comparisons with the reference group in class [5]. Feelings of deprivation and incompetence when comparing their performance with other peers might relate to feelings of lowered self-esteem, inferiority and a worse social standing among other students in class, resulting in lower life satisfaction. So far, there are still unresolved questions about whether and how the average performance of the class does relate to students’ life satisfaction, or whether there is a differential effect or vulnerability for students with high or low performance when placed in classes with on average higher performance. There is some evidence from original work on the BFLPE, which examined whether the size of the BFLPE differ for more and less able students in relation to their general or academic self-concept [8]. However, these studies showed mixed results and mainly small or insignificant interaction effects. Trautwein et al. [10], for instance, found a statistically significant positive interaction between school-average achievement and individual achievement, suggesting that high-achieving students were less affected by the negative frame of reference effect than were low-achieving students. In contrast, Marsh et al. [46] tested interaction effects between school-average ability and individual ability in two samples of college-track high school students. Whereas there was no evidence for an interaction effect in the first sample, a negative interaction term in the second sample suggested that high-achieving students were more strongly affected by placement in high-achieving schools.

Further, with regard to the differential impact of class-level performance, there is evidence from educational research, particularly from research on educational effectiveness, on the differential impact of the overall performance in class. Those studies indicated that students belonging to classes with higher performance levels benefit in terms of individual academic achievement, whereas the opposite holds true for students placed in low ability classes [47,48,49]. In this context, findings showed that classes with high performance levels are often characterized by a better and conducive class climate [45], better teacher-student relationships, higher teacher support and teaching quality [42,50,51]. In addition, according to school effectiveness research, low-performing students being placed in classes with on average low school performance are often faced with a loss of motivation and interest regarding school work [51], while embedded in learning environments which are characterized by more negative features of social climate, poorer teacher-student relationships, lower levels of teaching quality and general expectations towards schoolwork [42,50]. The overall learning climate in class is also related to students’ level of life satisfaction [52], by highlighting that higher teachers’ care and monitoring at class-level was negatively related to lower life satisfaction, while the individual perceived class climate was more strongly associated with life satisfaction. However, there is, to date, no study available which investigated associations between compositional characteristics of school classes—measured at the class-level—, for instance in terms of average performance of classes, and young people’s life satisfaction [53].

### 1.4. The Current Study and Hypotheses

Given the centrality of peers and classmates in adolescence, social comparison processes with the reference group of classmates and their performance are likely to be related to individual students’ life satisfaction. For instance, comparisons with other classmates, who are less favorably situated and gifted in terms of school performance, enhance the feeling of individual ability and competence, likely to result in higher life satisfaction. Furthermore, it is plausible that this association might be differential, depending on the individual students’ performance. 

The present study examines whether the average performance of classes is related to students’ life satisfaction, independently and in interaction with students’ individual performance. In line with the contrast- or BFLP-effect-hypothesis, we assume that students with comparable average individual performance, attending classes with on average higher performance, report lower life satisfaction (hypothesis 1). This hypothesis reflects the BFLPE-thesis in relation to students’ life satisfaction. 

In addition, we further investigate whether the average performance of the class is differentially associated with life satisfaction among students with high vs. low individual performance. We assume that low-performing students, attending classes with on average higher performance, may benefit from a more conducive learning environment, teacher and classmate support and a more nourishing and supportive class climate, and therefore show higher life satisfaction (hypothesis 2). In contrast, we assume for high-performing students, who are placed in classrooms with on average higher school performance, to be confronted with permanent comparisons and competition among classmates, which might be associated with lower life satisfaction for these students (hypothesis 3).

## 2. Materials and Methods 

### 2.1. Data

The National Educational Panel Study (NEPS) is carried out by the Leibniz Institute for Educational Trajectories at the University of Bamberg. It examines educational processes in Germany across the entire lifespan and is part of the “Framework Program for the Promotion of Empirical Educational Research” funded by the German Ministry of Education and Research and supported by federal states. NEPS started in 2010 with six starting cohorts (SC), each followed up annually or biannually [54] and offers an abundance of variables especially in the area of competence development, learning environments, educational decisions, migration background and educational returns [54].

This study focuses on individual measures from seventh graders (mostly aged 12 to 13 years, mean = 12.5 years, standard deviation = 0.6) in secondary regular schools in Germany, surveyed in wave 3 of the NEPS SC3 in 2012/2013. In total, the sample contains N = 6838 students, nested in 710 classes, which are nested in 277 schools. A further sample of 442 students in schools for students with special educational needs was excluded, due to insufficient comparability with questionnaires used for students in regular schools.

The sampling process followed a multi-step, stratified design. In the first step, schools were sampled from a comprehensive list of all schools in Germany. Data on school type and region were used for stratification of this sample, to replace non-participating schools with draws of similar schools, as the declared aim of NEPS is to provide a representative sample of the German educational landscape. In the second step, two or more classes from each participating schools were sampled. All students within these classes were asked to participate, as well as class, math and German teachers and students’ parents [55,56].

Students were interviewed in class by interviewers using paper and pencil interviews. The survey documents used were previously submitted to as well as reviewed and approved by the respective Ministries of Education of the 16 German federal states. During the survey, NEPS worked closely with the relevant data protection officers of the federal states for strict compliance with the statutory data protection regulations [57]. Ethical approval was obtained by the National Educational Panel Study (NEPS) from the ethical review boards of the 16 German federal states. This paper uses data from the National Educational Panel Study (NEPS): Starting Cohort Grade 5, doi:10.5157/NEPS:SC3:5.0.0. From 2008 to 2013, NEPS data was collected as part of the Framework Program for the Promotion of Empirical Educational Research funded by the German Federal Ministry of Education and Research (BMBF). As of 2014, NEPS is carried out by the Leibniz Institute for Educational Trajectories (LIfBi) at the University of Bamberg in cooperation with a nationwide network. The authors of this study only conducted secondary data analyses on these data and did not obtain any data on their own.

### 2.2. Indicators

#### 2.2.1. Life Satisfaction 

Subjective well-being was measured by life satisfaction. In general, well-being is a multi-dimensional construct, which can include either cognitive judgments, such as life satisfaction or positive emotions [24,29]. Life satisfaction was measured by asking students “how satisfied are you with your life?” (0 = ”not at all”–10 = “very satisfied”) [58]. The single-item measurements of life satisfaction have been shown to perform very similarly to the psychometrically established Satisfaction With Life Scale (SWLS) [59], and has been established as an important indicator in several studies on young people’s well-being [37,60]. It was used as a metric measure in our analyses.

#### 2.2.2. School Performance and Average Performance of the Class

School performance or just performance is used as a measure for students’ academic achievement in terms of school grades. In surveys with school-aged children, self-reports of school grades are often used because of their ease of response and high correlates students’ actual grade point averages (GPA) as shown in validation studies [61,62,63]. Performance was measured by their self-reported GPA which was calculated by using two major school subjects (i.e., Mathematics and German language) at the end of the school year. Both school subjects are obligatory for school-aged children in lower secondary education and students have to jointly attend those classes, which is a relevant precondition when examining the role of class-level composition on student outcomes. Students were asked “*What grade did you have on your last annual report card in… [German/Mathematics]?*” Response options were “1 = very good” to “6 = unsatisfactory”, indicating that lower grades correspond to better performance in the German grading system. For ease of interpretation and comparison with other international grading systems, both grades have been inverted (i.e., 1 = unsatisfactory to 6 = very good). An index averaging the school grades in Mathematics and German language was then created (mean = 4.32, standard deviation = 0.79, min = 1, max = 6, Pearson’s r = 0.48, Cronbach’s alpha: 0.66). At class-level, the average performance of the class (mean = 4.32, standard deviation = 0.42; min = 2, max = 5.5) is the aggregate of the classes’ students’ individual performances. The analyses use the z-standardized indicator for individual performance (mean = 0, standard deviation = 1) and class-mean of this indicator for class-average performance (mean = 0, standard deviation = 0.52).

#### 2.2.3. School Type

School type in the German school system is hierarchically organized, with co-existing different tracks in secondary education. Most commonly distinguished are the highest track “Gymnasium”, the intermediate track “Realschule”, the lowest track “Hauptschule”, and a comprehensive track “Gesamtschule”, combining aspects of all. All these track cover lower secondary education, but only the highest track “Gymnasium” and distinct tracks within the comprehensive track “Gesamtschule” also cover higher secondary education and the attainment of a university entrance qualification. NEPS provides data to partially distinguish the specific track students at the “Gesamtschule” attended and where possible, the students were categorized accordingly. Data on the school type is available from the NEPS sampling process. 

#### 2.2.4. Age and Gender

We controlled for age in years, which was centered for the analyses and also controlled for gender, using boys as the reference category. The middle 99%-interval for age was 11 to 14 years, due to 14 outliers which were addressed in the sensitivity analyses this range extends to 10 to 17 years for the full sample.

### 2.3. Sample

Table 1 presents the sample description and distribution of all indicators that have been used in this study. The mean age of the seventh graders is 12.5 years (standard deviation = 0.6), 49.6% of them are female, 53.3% visit the highest school track in Germany (“Gymnasium”), and the mean performance (measured via the average of grades in math and German) is 4.3, which lies between a “good” (grade 5) and “fair” (grade 4) evaluation in the German grading system. Due to missing data and the exclusion of classes for which less than 5 realized student interviews were available, the final analyses were performed on a reduced sample of n = 5196 students with complete data in 478 classes, nested in 250 schools. 

### 2.4. Statistical Analyses

For descriptive analyses, the study investigates correlations between students’ life satisfaction and their performance, the average performance of the class, and the differences thereof. For the multivariate analyses, it utilizes three-level multilevel analysis that allows the modelling of hierarchical or nested data structures. The level 1-units in the sample are individual students; the level 2-units are classes and level 3-units are schools. In this study, random intercept models are conducted to examine the variation of the outcome’s intercept among classrooms and schools [64]—a technique which has been applied by the majority of studies investigating compositional characteristics at the class-level [65].

In a stepwise approach, individual- and class-level indicators have been introduced to the models. The school-level was considered to adjust for the variation in the outcome that might be explained among schools. Model 0 is the empty model and tested the Intraclass Correlation Coefficient (ICC), which represents the proportion of variance on latent school and classroom effects by indicating the variance in the outcome attributed to differences between schools and classes. Model 1 tested for age, gender, and school type. Model 2 then included the individual performance as a metric z-standardized variable. In order to test the BFLPE (hypothesis 1), Model 3 added the average performance of the class, by also using the metric z-standardized variable of students’ individual performance. Model 4 then introduces a cross-level interaction term between the individual performance and the class-level average performance. The statistical analyses were conducted using the software R version 3.3.3 [66] and the package “lme4” for multilevel analyses [67].

The cross-level interaction included in the final model was further examined using simple slopes and—if indicated—an analysis of regions of significance [68]. The robustness of findings was further tested with a series of sensitivity analyses. Due to the outliers in student’s age, Models 0 to 4 were also tested after the exclusion of a total of 14 students aged either below 11 or above 14 years old. To examine the robustness of results, the final model was also tested on several subsets of the data (see Supplementary Material, Tables S1 and S2).

## 3. Results

### 3.1. Descriptive Results

Overall, Table 1 highlights that students report a high life satisfaction (mean = 7.47, standard deviation = 2.20) and good to fair performance (mean = 4.32, standard deviation = 0.79). When split by the median, 2309 students showed low, and 2887 students showed high performance. Table S3 (Supplementary Material) provides pairwise correlations of all variables used in the final model. Performance is positively correlated to the average performance of the class (*r* = 0.52, *p* < 0.001).

### 3.2. Multivariate Results

Table 2 shows the results from the linear multilevel models for life satisfaction. According to the Intraclass-Correlation Coefficients between schools (ICC < 0.1%) and between classes (ICC = 4.6%) in the empty model (Model 0), the variation of the outcome measure is mainly located at the individual-level. Due to the fact that there are very few classes sampled per school and thus both units of clustering are very similar, the ICC for at least one is likely to drop close to zero.

Model 1 only includes the controls and shows associations for gender and school type with life satisfaction. Overall, girls as well as students not visiting highest school track report significantly lower life satisfaction.

Adjusting for control variables, Model 2 considers students’ individual performance, being positively associated with life satisfaction, meaning that lower performance was related to lower life satisfaction. The class average school performance introduced in Model 2 is not significantly associated with life satisfaction, but the association with individual school performance remains unchanged. Comparing only Model 2 and Model 3, life satisfaction is attributable to individual school performance, whereas class composition was less strongly associated with life satisfaction.

Model 3 examines the class-level performance in addition to the individual performance. The class-level performance shows no significant association with life satisfaction, while other coefficients and results are virtually unchanged, when compared to Model 2.

Model 4 adds a cross-level interaction term between students’ individual performance and the average performance of the class to examine whether the performance-related environment in class is differentially related to young people’s life satisfaction. As before, the class-level performance shows no significant association with life satisfaction, but the cross-level interaction term does. This interaction is examined in more detail with simple slopes and also visualized below. Table 3 provides an analysis of simple slopes for the joint associations of individual performance and class-level performance with life satisfaction. For examination, the associations are shown for individual performance and class-level performance. They were set at the mean and one standard deviation below and above average performance [68]. The association between individual performance and life satisfaction is always significant and high-performing students show better life satisfaction than low-performing students. The results however indicate that for high-performing students there is also a significant association between class-level performance and life satisfaction. High-performing students show higher life satisfaction when placed in lower performing classes, than other high-performing students placed in higher performing classes. An analysis of the regions of significance for the association between class-level performance with life satisfaction with respect to individual performance show a lower threshold of −1.82 and an upper threshold of 0.55. Students with individual performance outside of these bounds show a significant association of the class-level performance with life performance.

**Figure 1:**
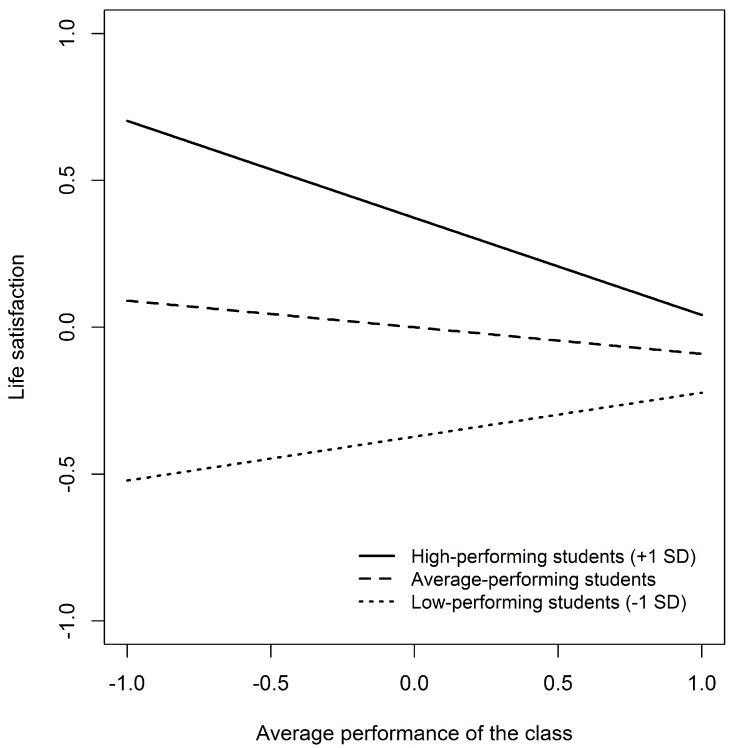
Predicted differences in school-aged children’s life satisfaction depending on individual performance and class-level average performance. Legend: Slopes from Model 4 for students at average performance and those students one standard deviation (SD) above as well as one standard deviation below students’ average performance. Higher values for the class-level average performance indicate higher-performing classes and vice versa. Life satisfaction is on a 0–10 metric and the figure shows differences in life satisfaction. The mean performance on the metric scale of 1 (“unsatisfactory”) to 6 (“very good”) was 4.32 (SD = 0.79). The figure was created with R [66].

Figure 1 visualizes the cross-level interaction term. It shows differences in life satisfaction on the y-axis depending on class-level average performance on the x-axis for high- and low-performing students. According to Figure 1 and the examination of the simple slopes, high-performing students show significant differences in life satisfaction depending on class-level average performance. Students with high performance show significantly lower life satisfaction when placed in high-performing classes, and higher life satisfaction when placed in low-performing classes. 

Sensitivity analyses of M0 to M4 after the exclusions of 14 outliers regarding students aged below 11 or above 14 years provided virtually identical results to those shown in Table 2. Further sensitivity analyses on M4 (see Supplementary Material, Tables S2 and S3) revealed mixed results, indicating that differential associations may exist, relating to class size and gender. The cross-level-interaction including the same pattern when examining simple slopes could only be reproduced for high track schools when splitting by school types (see models S1a and S1b in Tables S1 and S2) and for boys when splitting by gender (see models S3a and S3b in Tables S1 and S2).

## 4. Discussion

This study aimed at investigating whether and how average performance in classes is related to students’ life satisfaction, independently and in interaction with the students’ individual performance. In relation to studies on the BFLPE- or contrast-effect in educational settings, studies have highlighted, so far, that students with comparable performance placed in classes with higher average performance reported lower general and academic self-concept [69]. However, regarding students’ overall well-being in terms of life satisfaction, there are still unresolved questions about whether and how the class-level average performance does relate to students’ life satisfaction.

While schools are instrumental in the promotion of students’ overall well-being, it is vital to understand the impact that the learning environment in classrooms might have on positive youth development. Particularly early adolescence is a vulnerable stage in life, characterized by increased needs for autonomy as well as a positive self-evaluation [3]. It is also a period when comparisons with peers are likely to increase and reference group mechanisms in relation to comparisons of social and scholastic issues, such as school performance, may be enforced. According to the stage-environment-fit theory, mismatches between these needs and the characteristics of the learning context are likely to contribute to poor adjustment and low overall well-being among young people [70,71]. In addition, this study extended prior research on the contrast- or “big-fish-little-pond”-effect by examining whether and how life satisfaction of high- vs. low-performing students is affected when they are placed in classes with on average higher performance. Prior studies have not yet focused on students’ life satisfaction, but rather on self-concept in general—often measured by various indicators [7]–, and thus it is hardly possible to directly compare our findings with previous evidence.

Regarding hypothesis 1, findings do not support the BFLPE for life satisfaction, as it is defined as a class-level effect, independent of individual average performance or achievement. In other words, the average performance of the class is not associated with students’ life satisfaction, independently of their individual average performance. But the findings for the simple slopes between individual performance and the average performance of the class are indications for a differential sensitivity to class-level higher performance for low- vs. high-performing students.

This leads to hypotheses 2 and 3. Our findings do not support hypothesis 2, as students with lower individual performance did not show higher life satisfaction, when placed in classes with on average higher performance. Although the simple slope between lower individual performance and higher class-level average performance was not statistical significant, this finding is quite surprising from the viewpoint of social comparison theory which suggests that young people self-evaluate their own performance more positively when comparing themselves with also low-performing classmates, but more negatively when comparing them with better performing peers [2]. However, drawing on research on scholastic outcomes, for example, studies highlighted in this regard that students with poor performance benefit from better-performing classmates due to a more conducive learning environment, teacher and classmate support and a more nourishing and supportive class climate [3,72].

With regard to hypothesis 3, we further assumed that high-performing students placed in classrooms with on average higher school performance (hypothesis 3) are likely to report lower life satisfaction. Results confirm this hypothesis (Figure 1) and are in line with a study by Marsh and colleagues [46]. In contrast to this, two studies examined the school well-being of secondary school students in Belgium and tested students’ school well-being by asking students about their liking of the school [73,74]. In contrast to our findings, both studies concluded that the average achievement level of the class had an additional positive effect on students’ school well-being over and above the initial individual achievement of the students. In other words, these studies revealed that it is likely to be positively related to students’ school well-being when placed in a class with on average high-achieving classmates [7]. According to social comparison theories [69], high performing students in a homogeneous high-performing class are more likely to face constant reminders of their inadequacies when compared with their equally high-performing or even higher performing classmates, which is likely to be related to insecurity about their own school performance [7]. Further, this association is quite comprehensible as the overall learning environment in high-performing classes may be characterized by high academic effort and school engagement [75]. Therefore, it is likely that students require effort from other peers in relation to schoolwork, which challenges students’ psychological needs for competence and relatedness and puts pressure on those students [76,77]. In this context, it is plausible that students with higher school performance, being surrounded by also high-performing classmates, permanently compare themselves to counterparts in class who perform also well, notice higher levels of school requirements by peers and teachers and are likely to attempt to catch up and perform as well as their classmates, which is likely to be related to lower life satisfaction.

### Strengths and Limitations

First and foremost, our study only provides indications for a social comparison at work, as the variables used do not explicitly reflect the process of comparison with classmates. Second, although our study was based on a very strong and representative data set, some potential limitations should be addressed in future research on negative frame-of-reference-effects on students’ outcomes of well-being, such as life satisfaction. As this study was non-experimental and used cross-sectional data, causal interpretations require caution. The final sample showed a high proportion of missing data with 24% of cases lost. These drop outs are slightly skewed, as students attending low track schools are more likely to drop out than those in high track schools. Fourth, it is important to note that the majority of studies on the contrast- or “big-fish-little-pond”-hypothesis used “objective” measures (i.e., a standardized achievement test) of school performance, captured on a common metric [9,19]. This evidence showed, for instance, that academic self-concept is more related to class marks than standardized test scores [60]. Also, the study relies on a single-item measurement for life satisfaction and a two-item measurement for performance, which do provide less reliable measurements. Further, in relation to our dependent variable “life satisfaction”, studies showed that higher school performance—measured by subjective indicators of self-evaluated or perceived academic performance—is more strongly correlated with higher life satisfaction, whereas the association between objective measures of academic achievement (i.e., achievement test scores) and life satisfaction is less clear [41]. Based on these findings, we therefore decided to use self-reported grades instead of objective measures (i.e., test scores), while it also allowed for easier comparison of students with each other. However, in other surveys of child and adolescent health, such as the Health Behaviour in School-aged Children (HBSC)-Study [37], school performance was also measured by students’ self-report. A validation study [78] of this HBSC measure highlighted, that self-reported school performance highly correlates with students’ self-reported measure of school grades in different countries. Further, other studies, mainly from the US and a meta-analysis tested the validity of self-reported grade point averages of undergraduate or high school students. They found that correlations between self-reported grade point average and actual grade point average were very high (*r* > 0.70), did not differ largely between females and males or by parental education level, but were greater for white than for nonwhite students. Another study also found a high match between students’ self-reported grade point average and school-reported (i.e., more objective) measures on grade point average, while students’ indication of grade point average was more likely to be lower than the school-reported grade point average.

## 5. Conclusions

Schools are pivotal for young people’s psychosocial development as they spend most of their daily time at school and with classmates of different ability levels. This study adds further evidence on the association between students’ individual school performance and life satisfaction. Also, the role of average school performance in classrooms for young people’s life satisfaction has been examined. Finally, we further investigated whether students with different levels of school performance (i.e., high vs. low school performance) are associated with a differential vulnerability or sensitivity in life satisfaction when placed in classrooms with on average higher school performance. By doing this, it is possible to detect young people at risk, which could provide information, for instance, for teachers and school principals to arrange teaching in class, to compose learning groups within classes and to target school initiatives for vulnerable students.

This study investigated the so-called “big-fish-little-pond”-effect by examining the impact of class-level performance on life satisfaction. Our multilevel results did not support this effect, but instead showed that the association of class-level average performance with students’ life satisfaction varied between students with high vs. low performance. Our results showed that being placed in an on average high-performing class was not related to life satisfaction for students who report low school performance, but showed a negative association with life satisfaction among high-performing students. Being placed in a high performing class is characterized by a high share of classmates who are also very good at school and are also likely to be characterized as ambitious students. Since there is always some heterogeneity in class composition and the proportion of low- vs. high-performing students does not allow for all low-performing students to simply be placed in high-performing classes, results of this study suggest that it may be better for students to be grouped in classes of evenly distributed performance to avoid profuse upward- or downward-directed comparisons with classmates, which could promote interaction between students with different performance-levels. This indicates a need for school initiatives, undertaken for example by school psychologists and teachers, which emphasize not only students’ academic performances, but also their health and well-being. This could include, for instance, (a) schools and teachers are requested, to facilitate within-class grouping, aiming not only at grouping students by similar performance levels, but rather at mixing students with different performance-levels in heterogeneous learning groups within classes. Thereby, students with high school performance could notice that there are other classmates in their group who are also high performers, while students with higher school performance could offer help and support to their (e.g., poorer performing) peers. Further, (b) teachers and school psychologists can also attempt to strengthen students’ self-confidence and academic competences by focusing on their personal and social strengths and resources. It is likely that school performances play an important role in shaping relationships between teachers and students as well as relationships between students. Thus, teachers are required to establish positive learning environments which are characterized by a culture of acceptance and where students are perceived as individuals with their variety of social and emotional personality traits, rather than a learning culture that is shaped by a strong focus only on students’ academic performance and scholastic development. Or, (c) school initiatives could focus on students’ overall well-being, by not only emphasizing risk factors, such as school requirements, but also taking into account students’ particular strengths and abilities. This may of high importance as school is compulsory at least up to the tenth grade (in Germany) which makes it even more important to ensure that the learning environment in classrooms serves to enhance the overall well-being of all students.

## Figures and Tables

**Table 1:** Sample description (NEPS SC3, n = 5196 students).

	Frequencies	
	Absolute (n)	Relative (%)
Gender		
Boy	2617	50.4%
Girl	2579	49.6%
School type		
High track (“Gymnasium”)	2768	53.3%
Medium track (“Realschule”)	1501	28.9%
Mixed track (“Gesamtschule”)	530	10.2%
Low track (“Hauptschule”)	397	7.6%
Individual performance ^1^		
High	2887	55.6%
Low	2309	44.4%
	Mean (SD)	Min.–Max.
Individual-level indicators		
Life satisfaction	7.47 (2.20)	0–10
Age	12.51 (0.62)	10–17^2^
Individual performance ^1^	4.32 (0.79)	1–6
Class-level indicator		
Average performance of class	4.32 (0.42)	2.0–5.5

^1^ Performance was measured by calculating students’ individual means of school grades in Mathematics and German language. Presented are scores from the German grading system, which have been inverted to 1 = unsatisfactory to 6 = very good. These scores were z-standardized for the analyses and a median split has been applied in order to represent high- and low-performing students. The mean (SD) performance was 3.63 (0.46) for 2309 low performing students and 4.91 (0.44) for 2887 high-performing students. ^2^ The wide range is due to some outliers and the middle 99%-interval of the age distribution ranges from 11–14.

**Table 2:** Linear mixed models for students’ life satisfaction (n = 5196 students, in 478 classes, in 250 schools).

	Model 0	Model 1	Model 2	Model 3	Model 4
	b (SE)	b (SE)	b (SE)	b (SE)	b (SE)
Intercept	7.46 (0.04) ***	7.80 (0.06) ***	7.72 (0.06) ***	7.74 (0.06) ***	7.79 (0.06) ***
Individual-level variables					
Age ^c^		−0.04 (0.05)	0.01 (0.05)	0.01 (0.05)	0.01 (0.05)
Gender^ref=boys^		−0.30 (0.06) ***	−0.32 (0.06) ***	−0.32 (0.06) ***	−0.32 (0.06) ***
School type^ref=high track (Gymnasium)^					
Intermediate track (Realschule)		−0.32 (0.08) ***	−0.14 (0.08)	−0.18 (0.09)	−0.18 (0.09)
Low track (Hauptschule)		−0.44 (0.12) ***	−0.27 (0.13) *	−0.33 (0.14) *	−0.29 (0.14) *
Mixed track (Gesamtschule)		−0.50 (0.13) ***	−0.32 (0.12) **	−0.35 (0.12) **	−0.36 (0.12) **
Performance ^1^			0.39 (0.03) ***	0.38 (0.03) ***	0.37 (0.03) ***
Class-level variables					
Average class-level performance ^2^				−0.10 (0.09)	−0.07 (0.09)
Cross-level interaction term					
Average class-level performance ^2^× performance ^1^					−0.19 (0.05) ***
N Students	5196	5196	5196	5196	5196
N classes	478	478	478	478	478
N schools	250	250	250	250	250
σ² classes	0.218	0.172	0.179	0.177	0.169
σ² schools	<0.001	<0.001	<0.001	<0.001	<0.001
σ² residual	4.514	4.500	4.377	4.378	4.375
Deviance (−2LL)	22,776.13 (df = 4)	22,737.17 (df = 9)	22, 657.18 (df = 10)	22, 659.43 (df = 11)	22,599.10 (df = 12)
ICC class-level	4.61%	3.68%	3.93%	3.89%	3.72%

Model 0: empty model; Model 1: M0 + age + gender + school type; Model 2: M1 + performance; Model 3: M2 + average performance of the class; Model 4: M3 + interaction term; ^c^ = variable is centered at mean = 0; ^1^ = individual performance operationalized as the z-standardized grade point average over German language and Math (mean = 0, standard deviation = 1); ^2^ = average class-level performance mean of all individual performances in a class (mean = 0, standard deviation = 0.52); ref = reference group; SE = Standard Error; LL = Log Likelihood; df = degrees of freedom; Significance level: * = *p* < 0.05, ** = *p* < 0.01, *** = *p* < 0.001.

**Table 3:** Simple slopes.

Average Individual Performance	Average Class Performance	Estimate	Standard Error	t Value	df	*p* Value
−1 (−1SD)	sst	0.116	0.108	1.076	224	<0.284
0 (mean)	sst	−0.070	0.087	−0.810	224	<0.419
1 (+1SD)	sst	−0.257	0.098	−2.621	224	<0.010 **
sst	−0.52 (−1SD)	0.469	0.043	11.017	4714	<0.001 ***
sst	0 (mean)	0.372	0.034	10.855	4714	<0.001 ***
sst	0.52 (+1SD)	0.275	0.047	5.835	4714	<0.001 ***

sst = simple slope test, the variable for which the simple slope was estimated; SD = Standard Deviation; df = Degrees of Freedom; Significance level: ** *p* < 0.01, *** *p* < 0.001.

## Supplementary Materials

The following are available online at http://www.mdpi.com/1660-4601/15/12/2750/s1. Table S1: Sensitivity analyses: Alternate models, Table S2: Sensitivity analyses: Simple slopes of alternate models, Table S3: Sample correlations (NEPS SC3, N=5196 students).
