# Supplementary material for: Class-Level School Performance and Life Satisfaction: Differential Sensitivity for Low- and High-Performing School-Aged Children
Source: Int J Environ Res Public Health. 2018 Dec 5;15(12):2750. doi: 10.3390/ijerph15122750 (PMC6313449; doi:10.3390/ijerph15122750)
Supplement: Supplementary file 1 [file ijerph-15-02750-s001.pdf]

Table S1. Sensitivity analyses: Alternate models

	Model S1a b (SE)	Model S1b b (SE)	Model S2a b (SE)	Model S2b b (SE)	Model S3a b (SE)	Model S3b b (SE)
Intercept	7.70 (0.08)***	7.63 (0.08)***	7.94 (0.15)***	7.83 (0.11)***	7.76 (0.07)***	7.50 (0.08)***
Individual-level variables						
Age[z]	−0.01 (0.07)	0.02 (0.07)	0.02 (0.12)	−0.03 (0.08)	0.07 (0.07)	−0.10 (0.08)
Gender[ref=boys]	−0.14 (0.08)	−0.52 (0.09)***	−0.62 (0.15)***	−0.25 (0.09)**	-	-
School type[ref=high track (Gymnasium)]						
Intermediate track (Realschule)	-	-	−0.39 (0.20)	−0.33 (0.37)*	−0.05 (0.12)	−0.35 (0.13)**
Low track (Hauptschule)	-	-	−0.28 (0.24)	−0.88 (0.16)**	−0.35 (0.17)*	−0.19 (0.21)
Mixed track (Gesamtschule)	-	-	−0.33 (0.21)	−0.32 (0.21)*	−0.11 (0.16)	−0.62 (0.17)***
Performance[1,z]	0.51 (0.05)***	0.46 (0.09)***	0.41 (0.11)***	0.50 (0.07)***	0.45 (0.06)***	0.49 (0.07)***
Class-level variables						
Average class-level performance[2,z]	−0.01 (0.15)	−0.09 (0.18)	0.21 (0.24)	−0.35 (0.19)	−0.16 (0.14)	−0.01 (0.16)
Cross-level interaction term						
Average class-level performance[2,z] x performance[1,z]	−0.47 (0.14)***	−0.23 (0.18)	−0.78 (0.23)***	0.03 (0.14)	−0.48 (0.12)***	−0.13 (0.13)
N Students	2,768	2,428	986	2,003	5,196	5,196
N classes[3]	224	254	109	166	478	478
N schools[3]	111	143	83	122	250	250
σ² classes	0.148	0.160	<0.001	0.201	0.102	0.308
σ² schools	<0.001	<0.001	0.020	<0.001	<0.001	<0.001
σ² residual	3.903	5.002	5.054	4.159	4.314	4.440
Deviance (−2x LL)	11719.59 (df=9)	10889.5 (df=9)	4423.98 (df=12)	8621.644 (df=12)	11322.48 (df=11)	11319.23 (df=11)
ICC class-level	3.79%	3.20%	<0.01%	4.83%	2.36%	6.94%
ICC school-level	<0.01%	<0.01%	0.40%	<0.01%	<0.01%	<0.01%

Model S1a: only highest school track; Model S1b: only intermediate, low, and comprehensive school track; Model S2a: only classes with 24 or less students; Model S2b: only classes with 25 or more students; Model S3a: only boys; Model S3b: only girls; [z] = variable is z-standardized (mean=0, SD=1); [1] = individual performance operationalized as the z-standardized (mean=0, SD=1) grade point average over German language and Math; [2] = average class-level performance mean of all individual performances in a class; [3] = sums of number of classes and schools differ due to students with different tracks in the same classes or missing data on the number of students per class; SE = Standard Error; LL = Log Likelihood; Significance level: * $p < .05$, ** $p < 0.01$, *** $p < 0.001$.

Table S2. Sensitivity analyses: Simple slopes of alternate models

	Average performance	Average class peformance	Estimate	Standard Error	t value	df	P value
Model S1a	−0.73 (−1 SD)		0.200	0.144	1.388	112	0.168
	0.25 (mean)		−0.078	0.109	−0.717	112	0.475
	1.22 (+1 SD)		−0.355	0.126	−2.814	112	<0.006**
		−0.22 (−1 SD)	0.461	0.054	8.557	2540	<0.001***
		0.25 (mean)	0.329	0.044	7.431	2540	<0.001***
		0.71 (+1 SD)	0.197	0.063	3.123	2540	<0.01**
Model S1b	−1.23 (−1 SD)		0.090	0.177	0.507	110	0.613
	−0.28 (mean)		−0.038	0.137	−0.28	110	0.780
	0.67 (+1 SD)		−0.167	0.168	−0.993	110	0.323
		−0.71 (−1 SD)	0.458	0.068	6.717	2170	<0.001***
		−0.28 (mean)	0.400	0.054	7.463	2170	<0.001***
		0.15 (+1 SD)	0.343	0.075	4.595	2170	<0.001***
Model S2a	−1.04 (−1 SD)		0.633	0.240	2.642	23	<0.015*
	−0.06 (mean)		0.178	0.184	0.968	23	0.343
	0.92 (+1 SD)		−0.276	0.217	−1.274	23	0.216
		−0.58 (−1 SD)	0.594	0.106	5.613	873	<0.001***
		−0.06 (mean)	0.356	0.087	4.102	873	<0.001***
		0.45 (+1 SD)	0.118	0.118	1.004	873	0.315
Model S2b	−0.98 (−1 SD)		−0.292	0.180	−1.619	43	0.113
	0.02 (mean)		−0.278	0.151	−1.845	43	0.072
	1.02 (+1 SD)		−0.264	0.168	−1.574	43	0.123
		−0.52 (−1 SD)	0.384	0.068	5.626	1833	<0.001***
		0.02 (mean)	0.392	0.054	7.24	1833	<0.001***
		0.55 (+1 SD)	0.399	0.074	5.358	1833	<0.001***
Model S3a	−1.07 (−1 SD)		0.190	0.143	1.325	212	0.187
	−0.04 (mean)		−0.111	0.111	−1.005	212	0.316
	0.98 (+1 SD)		−0.412	0.124	−3.334	212	<0.002**
		−0.54 (−1 SD)	0.509	0.057	8.948	2151	<0.001***
		−0.02 (mean)	0.357	0.046	7.72	2151	<0.001***
		0.50 (+1 SD)	0.204	0.063	3.25	2151	<0.01**
Model S3b	−0.93 (−1 SD)		0.066	0.152	0.436	205	0.663
	0.04 (mean)		−0.008	0.124	−0.061	205	0.951
	1.01 (+1 SD)		−0.082	0.141	−0.579	205	0.564
		−0.50 (−1 SD)	0.417	0.064	6.532	2122	<0.001***
		0.02 (mean)	0.377	0.052	7.288	2122	<0.001***
		0.54 (+1 SD)	0.338	0.070	4.835	2122	<0.001***

Model S1a: only highest school track; Model S1b: only intermediate, low, and comprehensive school track; Model S2a: only classes with 24 or less students; Model S2b: only classes with 25 or more students; Model S3a: only boys; Model S3b: only girls; SD = Standard Deviation; df = Degrees of Freedom; Significance level: * $p < 0.05$, ** $p < 0.01$, *** $p < 0.001$.

Table S3. Sample correlations (NEPS SC3, N=5,196 students)

	(1)	(2)	(3)	(4)	(5)	(6)	(7)	(8)
(1) Life satisfaction								
(2) Age	−0.03*							
(3) Gender (girl)	−0.07***	−0.09***						
(4) School type (high track)[a]	0.09***	−0.24***	0.03*					
(5) School type (medium track)[a]	−0.04**	0.12***	−0.03	−0.18***				
(6) School type (mixed track)[a]	−0.05**	0.07***	0.02	−0.10***	−0.21***			
(7) School type (low track)[a]	−0.04**	0.16***	−0.03*	−0.31***	−0.68***	−0.36***		
(8) Performance[b]	0.17***	−0.16***	0.04**	−0.13***	−0.20***	−0.03*	0.26***	
(9) Average performance of the class	0.09***	−0.18***	0.04**	−0.25***	−0.38***	−0.06***	0.51***	0.52***

Pearson's correlation coefficients and pairwise correlations. [a] The variable school type was transformed into four separate dummy-coded variables for this representation. [b] Performance was not dichotomized for this analysis. Significance level: * $p < 0.05$, ** $p < 0.01$, *** $p < 0.001$.
